# Pharmacokinetics and pharmacodynamics of intravenously and subcutaneously administered pantoprazole in sheep (*Ovis aries*)

**DOI:** 10.1371/journal.pone.0304533

**Published:** 2024-06-12

**Authors:** Joe S. Smith, Kailee Bennett, Ryan Flynn, Jesse Gebert, Pierre-Yves Mulon, Jessica D. Garcia, Lainey Harvill, Olivia Escher, Lisa Sams Ebner, Joan Bergman, Sherry Cox

**Affiliations:** 1 Large Animal Clinical Sciences, College of Veterinary Medicine, University of Tennessee, Knoxville, TN, United States of America; 2 Biomedical Sciences, College of Veterinary Medicine, Iowa State University, Ames, IA, United States of America; 3 College of Veterinary Medicine, Lincoln Memorial University, Harrogate, TN, United States of America; 4 Biomedical and Diagnostic Sciences, College of Veterinary Medicine, University of Tennessee, Knoxville, TN, United States of America; Acharya Nagarjuna University, INDIA

## Abstract

Abomasal ulcers are recognized in sheep of all ages, but research regarding therapeutic interventions is limited. Proton Pump Inhibitors (PPIs) such as pantoprazole, are clinically used with a paucity of evidence regarding efficacy in mature sheep. Intravenous and subcutaneously administered pantoprazole dosed at 1.0 mg/kg in adult sheep will increase the pH of abomasal fluid compared to pre-administration baseline. The objectives were to assess the effect of pantoprazole, after single and multiple administration, on abomasal fluid pH in adult sheep. A third objective was to describe the pharmacokinetic parameters of IV and SC pantoprazole. Four clinically healthy adult Southdown ewes previously fitted with a gastrostomy tube in the abomasum were utilized in this randomized, 2-way cross-over trial. Ewes received pantoprazole (1.0 mg/kg) as a single and 3-dose regimen (every 24 hours). After a 10 day washout period the reverse treatment was applied. Blood for analysis of pantoprazole concentration was collected intermittently for 24 hours, and abomasal fluid pH was measured at intervals for a 96-hour period. The pH of the abomasal fluid was higher in pantoprazole treatments for up to 24 hours after dosing. Following intravenous administration of pantoprazole to study ewes, elimination half-life, volume of distribution, and clearance of pantoprazole was estimated as 3.29 hours, 0.35 L/kg, and 65.26 mL/hr/kg respectively. After subcutaneous dosing, maximum concentration, time to maximum concentration, half-life of elimination, and volume of distribution, were estimated as 2604 ng/mL, 0.55 hours, 2.48 hours, and 0.37 L/kg. Additionally, the bioavailability was estimated as 83.33%. Pantoprazole administered IV or SC may be useful for treatment or prevention of abomasal ulcers in adult sheep.

## Introduction

Ulceration of the stomach lining is a complex disease that has been documented in many species [1–4]. In ruminants, abomasal ulceration is a common cause of morbidity and mortality described in cattle and sheep [1, 5–7]. Multiple factors are thought to contribute to gastric (abomasal) ulcers in ruminating species, and these factors include: stress, housing, nutritional imbalances, and non-steroidal anti-inflammatory drugs [1, 6]. Multiple pharmacologic agents are described for the treatment of gastric ulceration, with the proton pump inhibitor class thought to be the most efficacious of the gastroprotectants [4] due to the mechanism of action where the acid secreting molecular pump is irreversibly bound by the drug. Of the proton pump inhibitor drugs, one that is commonly used in veterinary medicine is pantoprazole.

The use of pantoprazole is described for multiple ruminating species, including alpacas, camels, yaks, cattle, goats, and sheep [8–13]. However, the pharmacokinetics of pantoprazole are less well described with reports existing for alpacas, goats and calves [8, 14–16]. Even less investigated is the pharmacodynamic effect of pantoprazole on abomasal pH, with descriptions in the literature only for alpacas and calves [8, 15]. As such, the clinical usage of pantoprazole in sheep is currently lacking support in the form of pharmacodynamic evaluation of the effects on abomasal pH. The goals of this study are to report the effect on intravenously (IV) or subcutaneously (SC) administered pantoprazole on the abomasal pH of adult ewes. It is hypothesized that similar to alpacas, administration of pantoprazole will lead to a statistically significant elevation of abomasal pH. An additional goal of the study was to report the pharmacokinetics of pantoprazole after IV and SC administration in sheep.

## Materials and methods

### Animals

Four, open Southdown-cross ewes, aged 2.75 ± 0.96 years of age and weighing 67.9 ± 7.6 kg were utilized for this study. The ewes had an abomasal cannula implanted 2 weeks prior as previously described for calves and ewes [15, 17]. Ewes were allowed to acclimate for one week upon arrival at the Veterinary Research and Education Center facility. Ewes were weighed on entry, deemed healthy by physical examination by a board-certified large animal specialist, and placed in individual screen pens that were adjacent. All ewes were fed an *ad libitum* grass hay diet, and had access to water *ad libitum*. All undertaken procedures were approved by the University of Tennessee Institutional Animal Care and Use Committee (IACUC #2835–0521). For all sampling events, ewes were fully conscious and restrained manually.

An intravenous jugular catheter (MILACATH®-EXTENDED USE, 16 Ga × 7.5 cm, MILA International Inc.) was placed aseptically (one in each vein for the IV component and one in the left vein for the SC component) 2 h prior to initiation of the 24-h study. For the IV study, one catheter was reserved for drug administration, and the second catheter was designated for sample collection. No drugs had been administered to the sheep for 18 days prior to the study. Pantoprazole (Pantoprazole Sodium for injection, Mylan International, Rockford Il) was administered at a 1.0 mg/kg dosage as a single dose (based on a described safe intravenous dose for sheep, goats and cattle) [13], with the dosing catheter flushed with 10 mL of 0.9% saline afterwards to ensure all drug was administered. The pantoprazole sodium was reconstituted with to a concentration of 4 mg/ml with 0.9% sodium chloride per manufacturer recommendations. The SC study utilized the skin of the neck, alternating from the left to right to left for each administration. The study utilized a random crossover design. Sheep were randomly assigned to receive pantoprazole IV or SC, then after a 10 day washout period the opposite treatment was administered. While the pharmacokinetic aspect of this study was for a 24 hour period after a single dose, the pharmacodynamic study investigated abomasal pH changes over a 3 dose (every 24 hr) period.

Blood samples were obtained from the designated sampling jugular catheter at 0, 5, 10, 20, 30, and 45 min after the first administration of pantoprazole by the IV and SC routes. Blood samples were also collected 1, 1.5, 2, 3, 4, 8, 12, 18, and 24 h after administration. Whole blood samples were collected via the push-pull technique from the designated catheter, with 4 mL blood placed into a lithium heparin tube (BD Vacutainer, BD) and immediately put on ice prior to centrifugation. Samples were spun down at 3,000 × g for 10 min and then plasma placed in cryovials (Cryogenic Vials, Biologix) and frozen at −80°C.

### Abomasal pH measurement

Abomasal fluid was collected via the abomasal cannula as described for calves and ewes [15, 17]. Fluid was collected each day of the study [day 0: pre-pantoprazole administration “control” and day 1–3 post-pantoprazole administration (q 24 hr)] using the same schedule: 0, 1, 2, 3, 4, 6, 8, 12, 18, and 24 h for both control samples as well as after esomeprazole administration. A 3 mm × 70 mm stainless steel teat cannula attached to a 12 mL syringe was introduced into the cannula past the unidirectional valve in the abomasal cannula. Abomasal fluid was aspirated via negative pressure using the 12 mL syringe until 4–5 mL of fluid was collected. The samples of abomasal fluid were placed into a 30 mL conical vial. A benchtop pH analyzer (UB-10 pH/mV meter, Denver Instruments, United States) was used to measure pH of the abomasal fluid samples. The pH meter was calibrated prior to each sample set according to the manufacturer’s recommendations. Once calibrated, the probe was introduced into the abomasal content sample and equilibrated for 30 s, which is when the pH level was recorded. The pH of all samples was recorded within 15 min of collection.

### Analytical chemistry

Determination of pantoprazole and pantoprazole sulfone concentrations from plasma samples was conducted as described for reverse phase high performance liquid chromatography (HPLC) goat plasma by Cox et al. [18]. The analytical system consisted of a 2,695 separations module and a 2,487 UV absorbance detector (Waters). The compounds were separated on a Symmetry C18 (4.6 × 150 mm, 5 μm) column with a 5 μm Symmetry C18 guard column. The mobile phase was a mixture of 0.1 M sodium phosphate dibasic and acetonitrile (68:32). The flow rate was 1 mL/min, and absorbance was measured at 290 nm.

Pantoprazole and its sulfone metabolite were extracted from the plasma samples using a liquid-liquid extraction method [18]. Samples were thawed, vortex-mixed, and 100 μl of plasma was transferred to a 13 × 100 mm screw top tube, followed by 10 μl of tinidazole (internal standard, 100 μg/mL) and 2 mL of chloroform. The tubes were rocked for 15 min and then centrifuged for 20 min at 1,000 × g. The organic layer was transferred to a glass tube and evaporated to dryness with nitrogen gas. Samples were reconstituted in 250 μL of mobile phase, and 100 μL was analyzed.

Standard curves for the plasma analysis were prepared by fortifying untreated, pooled plasma with pantoprazole and the sulfone metabolite, which produced a linear concentration range of 0.01–100 μg/mL. The average recovery for pantoprazole and its metabolite was 100 and 90%. The average recovery for the internal standard was 99%. The quality control (QC) samples used for validation were 0.03, 0.3, 3, and 30 μg/mL, and the intra and inter-assay variability was less than 11% for pantoprazole and for the metabolite. The lower limit of quantification for both was 0.1 μg/mL.

### Pharmacokinetic analysis

#### Pharmacokinetic analysis

As previously described for pantoprazole, pharmacokinetic analysis of total pantoprazole plasma concentrations was completed using a statistical moment (i.e., non-compartmental) approach in commercial software (PKanalix, Monolix Suite 2023R1, Lixoft, France) [14, 15]. Time versus concentration figures for pantoprazole were produced using a commercial software program (GraphPad Prism version 8.0, GraphPad Software, La Jolla California USA).

Standard PK parameters were generated for individual sheep, as follows:

- Maximum pantoprazole concentration after IV administration, **C0**;
- Maximum pantoprazole concentration after SC administration, **Cmax**;
- Time of maximum pantoprazole concentration after SC administration, **Tmax**;
- Area under pantoprazole concentration-time curve, **AUClast**, **AUCinf**:
- Area under the moment curve, **AUMCinf**;
- Pantoprazole mean residence time, **MRT;**

MRT = AUMCinf ⁄ AUC inf;

- Slope of the elimination phase **λ**_**z**_, computed by linear regression of the logarithmic concentration vs. time curve during the elimination phase;
- Pantoprazole terminal half-life, **T**_**1/2**_ **(λz);**
- T_1/2_ (λz) = ln (2) ⁄ λz;
- Pantoprazole systemic clearance, **CL;**
- Volume of distribution of pantoprazole after IV administration, **Vz**;
- Or apparent volume of distribution of pantoprazole after SC administration, **Vz/F**;
- Volume of distribution of pantoprazole estimated at steady-state, **Vss;**

**C**_**0**_ (Calculated concentration at time zero)**; Cmax** (Maximum plasma concentration); **Tmax** (Time of maximum plasma concentration); **AUClast** (Area under the concentration-time curve from time 0 to the last observable timepoint), **AUCinf** (Area under the concentration-time curve from time 0 to infinity), **AUMCinf** (Area under the moment curve from time 0 to infinity); **MRT** (Mean residence time); **λ**_**z**_ (Slope of the terminal (elimination) phase); **T**_**1/2**_ **(λz)** (Terminal (elimination) half-life); **CL** (Systemic clearance); **Vz** (Volume of distribution during the elimination phase); **Vz/F** (Apparent volume of distribution during the elimination phase); and **Vss** (Volume of distribution at steady-state).

A linear/log trapezoidal rule was used to estimate the area under the pantoprazole time-curves.

Selection of timepoints for determination of λz for each individual was performed automatically by the PKanalix 2021R1 software using the adjusted R2 method and checked manually prior to running the non-compartmental analysis. A minimum of 3 timepoints was selected for estimating the slope of the terminal phase. λz was calculated via a linear regression between Y = log(concentrations) and the X = time. The 1/Y^2^ weighting method was used for the regression analysis.

For pantoprazole sulfone, the following above parameters were reported: C_max_; T_max_; AUC_last_; AUC_inf_; MRT_inf_; and T_1/2(λz)_.

Summary statistics on the individual PK parameters were performed thereafter to derive the geometric mean, and (min-max) range. Elimination half-life was reported as harmonic mean.

### Statistical evaluation

Statistical evaluation of pH data was performed as reported by Olivarez et al. [15]. First data from pH values were evaluated for normality. Next, one-way ANOVA followed by multiple comparisons (utilizing Dunnett’s multiple comparisons test) was conducted using GraphPad Prism (version 8.0, GraphPad Software, La Jolla California USA). For this analysis day zero (no treatment) was the baseline being compared to individual pantoprazole treatment days. For this study, P values of < 0.05 were considered statistically significant.

## Results

### Animals

Abomasal cannulas were well-tolerated by all study ewes, with maintenance of appetite and body weight throughout the entire study period. Cannulas were removed 28 days after collection of the last samples. Follow up 5 months after the conclusion of the study demonstrated all ewes were in good body condition and were reported as doing well. Pantoprazole administration appeared to be well tolerated with no evidence of adverse reaction after being administered by the IV or SC routes.

### Abomasal pH measurement

**Fig 1:**
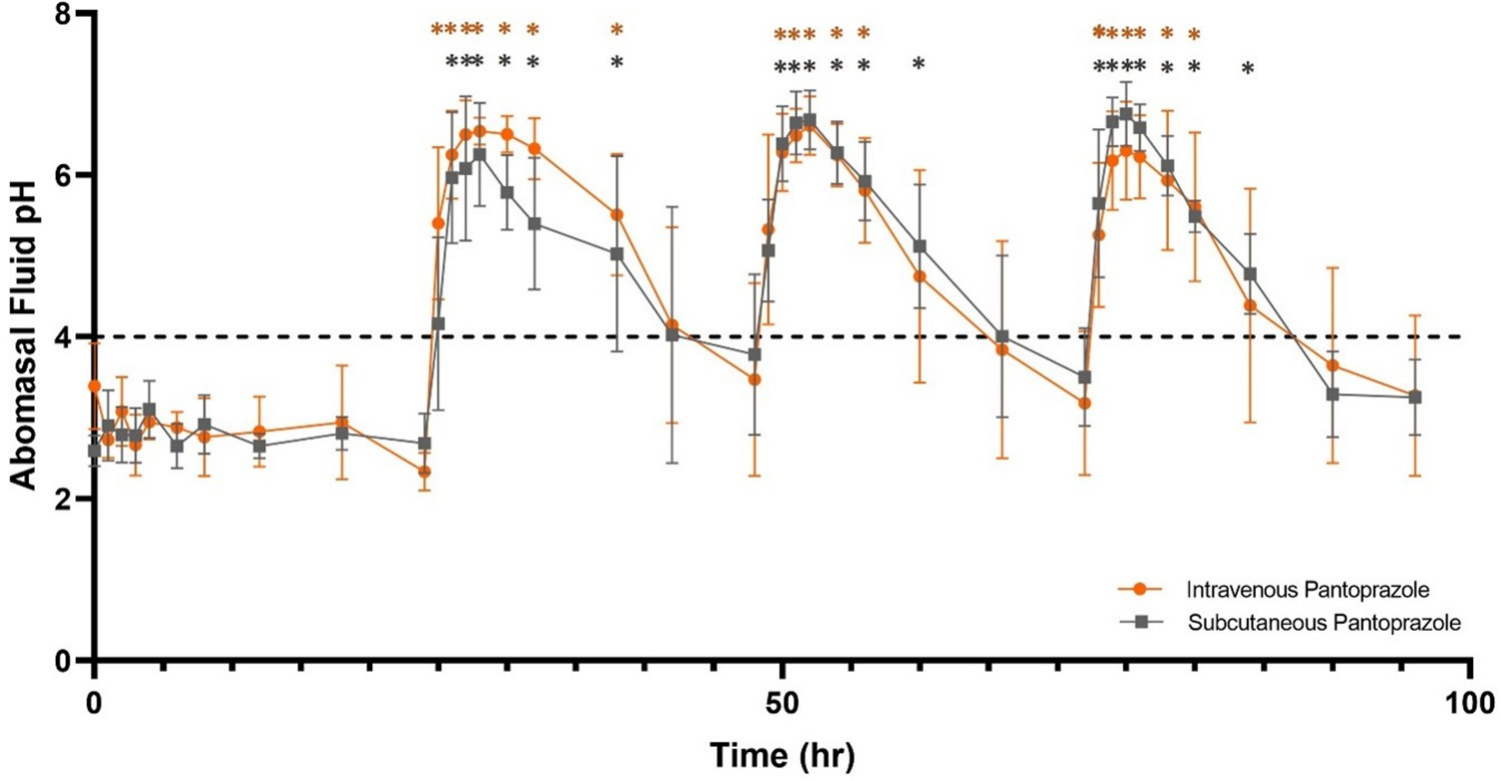
Abomasal pH of study ewes (n = 4) on day 0, as well as days 1–3 post pantoprazole (1 mg/kg) administration via the intravenous (IV: Orange) or subcutaneous (SC: Grey) routes. Asterisk (*) indicates statistically significant (P < 0.05) difference from pre-pantoprazole administration baseline. Dashed line represents a pH of 4.0.

Abomasal pH on day 0 (control) ranged from 2.29 ± 0.26 to 3.39 ± 0.46. After initial IV administration of pantoprazole, abomasal pH ranged from a peak of 6.54 ± 0.17 at 4 hours after administration to a low of 3.39 ± 0.46 at 24 h post-administration. After the second IV pantoprazole treatment, abomasal pH ranged from a peak of 6.61 ± 0.36 at 4 hours after administration to a low of 3.18 ± 0.90 at 24 h post-administration. After the third IV pantoprazole treatment, abomasal pH ranged from a peak of 6.30 ± 0.61 at 3 hours after administration to a low of 3.27 ± 0.99 at 24 h post-administration. Statistically significant increases in pH over baseline were observed from 1 to 12 h after administration (**Table 1**) for the first IV pantoprazole administration; from 2 to 8 hr after the second; and from 1 to 8 hr after the third administration. For all IV administrations the pH remained above 4.0 until at least the 12 hr timepoint after administration. **Fig 1** demonstrates the abomasal pH of study ewes throughout the course of the study.

**Table 1:** Abomasal pH values during various timepoints after administration of 1.0 mg/kg of pantoprazole intravenously once daily for 3 administrations in adult ewes. Hours 0–18 correspond to pre-administration baseline, and hours 24–42; 48–66; and 72–90 correspond to the first, second and third administrations respectfully.

Hr	pH	Hr	pH	P	Hr	pH	P	Hr	pH	P
0	3.3925	24	2.3325	0.0546	48	3.475	0.9031	72	3.1825	0.9994
1	2.7275	25	**5.405**	0.0254*	49	**5.3275**	0.0600	73	**5.26**	0.0412*
2	3.0775	26	**6.25**	0.0009*	50	**6.28**	0.0003*	74	**6.1775**	0.0003*
3	2.6625	27	**6.4975**	0.0041*	51	**6.485**	0.0007*	75	**6.3025**	0.0027*
4	2.9475	28	**6.54**	<0.0001*	52	**6.6075**	0.0003*	76	**6.2225**	0.0020*
6	2.8825	30	**6.5025**	0.0005*	54	**6.245**	0.0021*	78	**5.9325**	0.0198*
8	2.7625	32	**6.3275**	0.0039*	56	**5.81**	0.0182*	80	**5.605**	0.0353*
12	2.83	38	**5.51**	0.0253*	60	**4.7475**	0.1527	84	**4.3875**	0.2651
18	2.945	42	**4.145**	0.4312	66	3.8425	0.5923	90	3.645	0.6909

Values greater than 4 are bolded. Adjusted p values of less than 0.05 considered statistically significant(*).

Prior to SC administration abomasal pH on day 0 (control) ranged from 2.48 ± 0.19 to 3.15 ± 0.34. After initial SC administration of pantoprazole, abomasal pH ranged from a peak of 6.25 ± 0.64 at 4 hours after administration to a low of 3.78 ± 0.99 at 24 h post-administration. After the second SC pantoprazole treatment, abomasal pH ranged from a peak of 6.68 ± 0.37 at 4 hours after administration to a low of 3.50 ± 0.60 at 24 h post-administration. After the third SC pantoprazole treatment, abomasal pH ranged from a peak of 6.75 ± 0.39 at 3 hours after administration to a low of 3.25 ± 0.47 at 24 h post-administration. Statistically significant increases in pH over baseline were observed from 2 to 12 h after administration (**Table 2**) for the first SC pantoprazole administration; from 1 to 12 hr after the second; and from 1 to 12 hr after the third administration. For all SC administrations the pH remained above 4.0 until at least the 12 hr timepoint after administration, with pH still remaining above 4.0 after the 18 hr post administration after the first and second doses. **Fig 1** demonstrates the abomasal pH of study ewes throughout the course of the study.

**Table 2:** Abomasal pH values during various timepoints after administration of 1.0 mg/kg of pantoprazole subcutaneously once daily for 3 administrations in adult ewes. Hours 0–18 correspond to pre-administration baseline, and hours 24–42; 48–66; and 72–90 correspond to the first, second and third administrations respectfully.

Hr	pH	Hr	pH	P	Hr	pH	P	Hr	pH	P
0	2.5925	24	2.6875	0.9034	48	3.7825	0.1362	72	3.5025	0.0645
1	2.9075	25	**4.1625**	0.1154	49	**5.07**	0.0051*	73	**5.65**	0.0367*
2	2.79	26	**5.965**	0.0080*	50	**6.385**	0.0048*	74	**6.655**	0.0020*
3	2.785	27	**6.0825**	0.0096*	51	**6.645**	0.0036*	75	**6.7525**	0.0034*
4	3.105	28	**6.2525**	0.0020*	52	**6.68**	0.0024*	76	**6.5825**	0.0011*
6	2.65	30	**5.785**	0.0026*	54	**6.275**	0.0032*	78	**6.115**	0.0036*
8	2.92	32	**5.4**	0.0211*	56	**5.925**	0.0105*	80	**5.4875**	0.0039*
12	2.6525	38	**5.025**	0.0479*	60	**5.1175**	0.0171*	84	**4.7775**	0.0031*
18	2.81	42	**4.0225**	0.4596	66	**4.0075**	0.2331	90	3.2925	0.2983

Values greater than 4 are bolded. Adjusted P value less than 0.05 considered statistically significant(*).

### Pharmacokinetics

**Fig 2:**
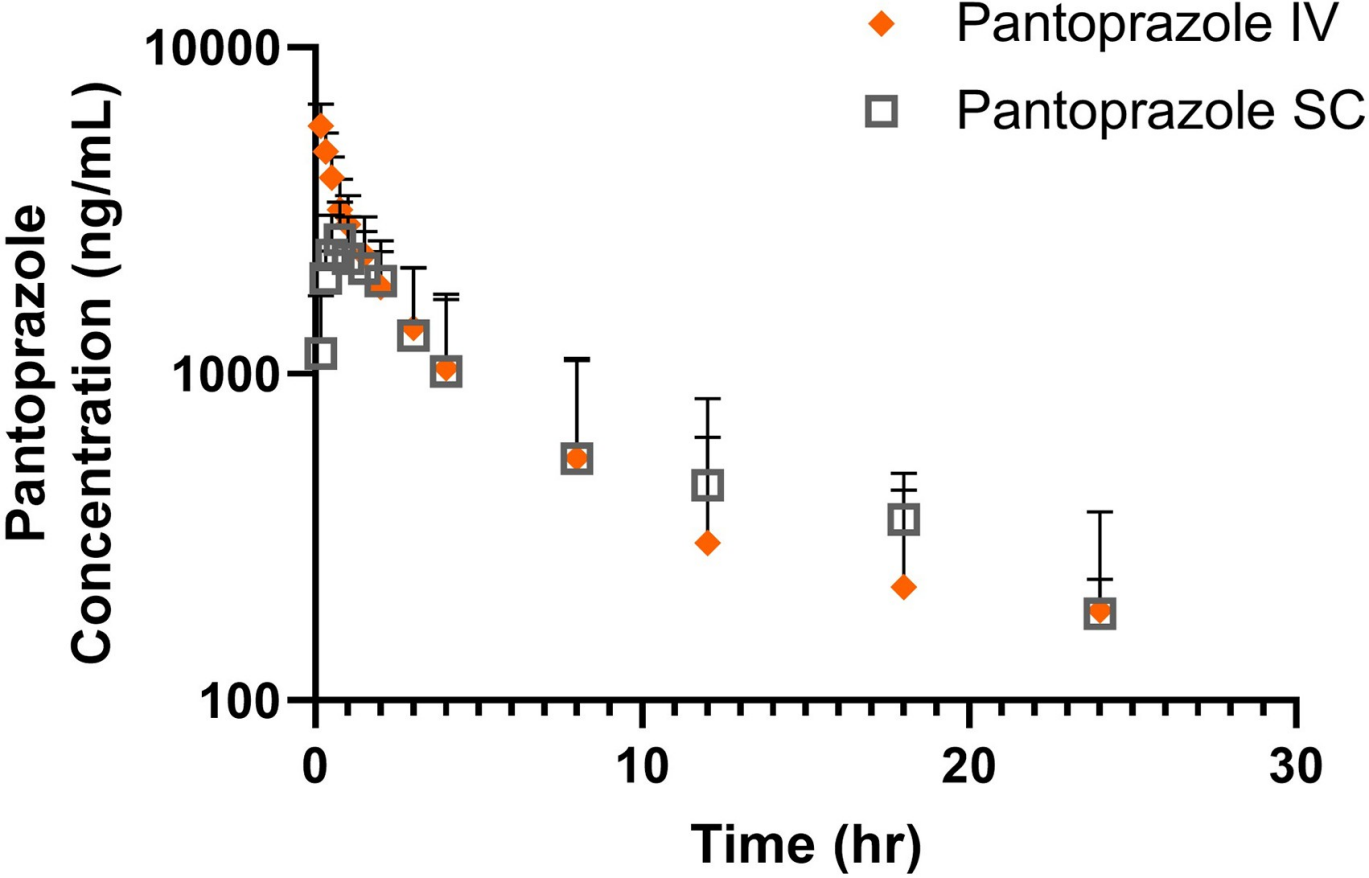
Time vs concentration curve for pantoprazole after intravenous (orange diamond) and subcutaneous (grey square) administration of 1.0 mg/kg to adult ewes. Upward bars represent error.

**Fig 3:**
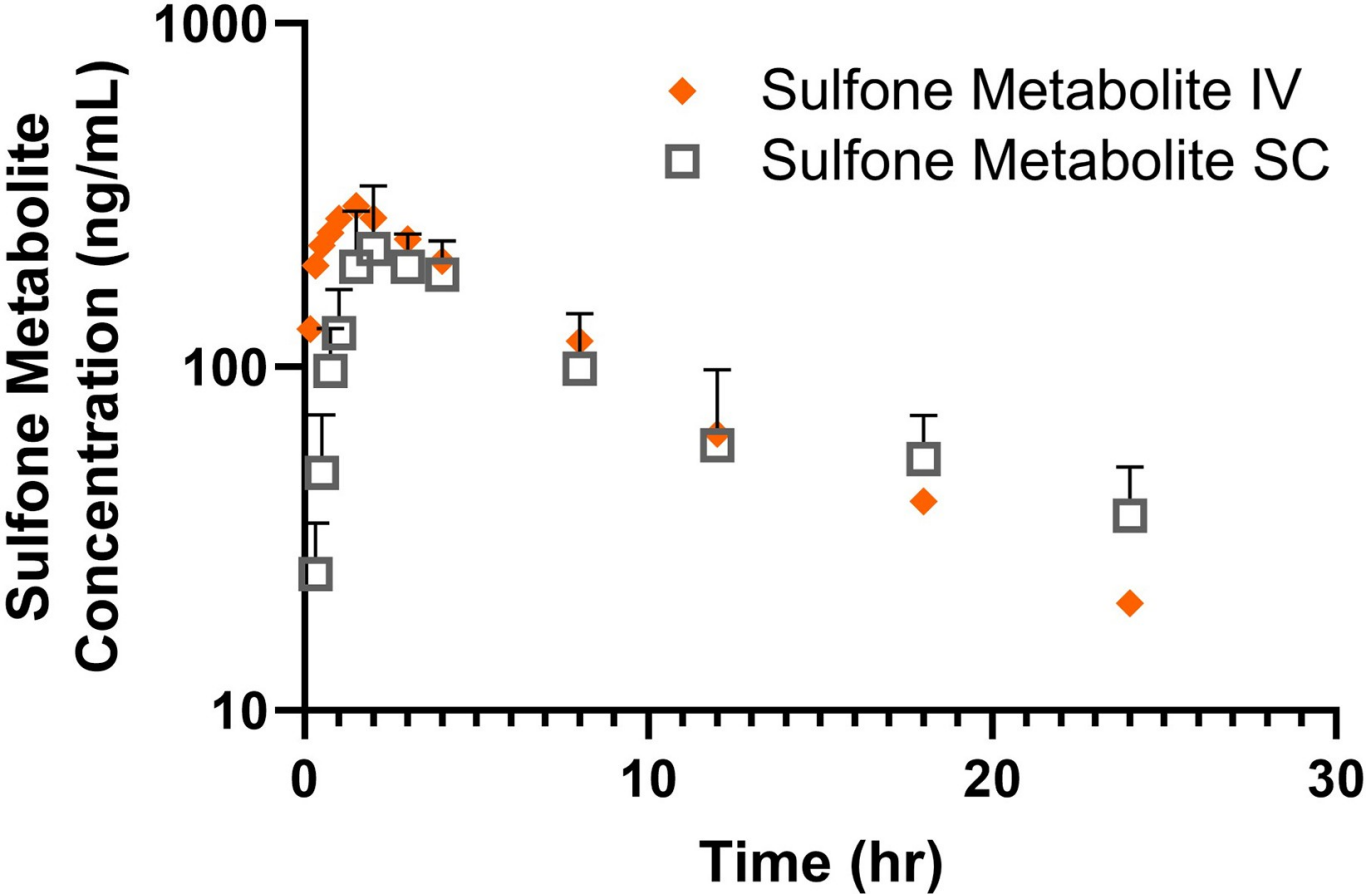
Time vs concentration curve for pantoprazole sulfone after intravenous (orange diamond) and subcutaneous (grey square) administration of 1.0 mg/kg pantoprazole sodium to adult ewes. Upward bars represent error.

No sheep had detectable pantoprazole in plasma at time zero. Geometric mean and standard deviations od pharmacokinetic parameters for pantoprazole and pantoprazole sulfone are presented in Tables 3 and 4 respectively. After administration, the parent drug and metabolite were detectable for up to 24 hr. After IV administration maximum (C0) concentrations were 6085 ± 2317 ng/mL for the pantoprazole parent drug and (Cmax) 281.5 ± 111.6 ng/mL for the sulfone metabolite. After SC administration maximum (Cmax) concentrations were 2604 ± 381 ng/mL for the pantoprazole parent drug and (Cmax) 229.3 ± 102.2 ng/mL for the sulfone metabolite Pertinent pharmacokinetic parameters are listed in **Tables 3 and 4**. **Fig 2** displays the time vs concentration curve for pantoprazole and **Fig 3** displays the time vs concentration curve for pantoprazole sulfone in the study ewes.

**Table 3:** Pharmacokinetic parameters of pantoprazole after single dose intravenous (IV; 1 mg/kg) and subcutaneous (SC; 1 mg/kg) administration in sheep (n = 4).

Compound (Route)	Parameter	Unit	Mean ± SD	Min	Max
Pantoprazole	C0	ng/mL	6085 ± 2317	4332	9249
(IV)	AUC_last_	hr*ng/mL	14.65 ± 9.523	9.09	29.78
	AUC_inf_	hr*ng/mL	15.23 ± 11.18	9.15	33.39
	AUMC_inf_	hr*ng/mL	64.65 ± 14.69	23.74	333.11
	MRT_inf_	hr	4.24 ± 3.5	2.32	9.98
	Cl	mL/hr/kg	65.26 ± 29.7	33.74	99.11
	T_1/2_ (λz)	hr	3.29 ± 2.59	1.95	7.78
	λz	1/hr	0.19 ± 0.12	0.09	0.35
	V_z_	L/kg	0.35 ± 0.06	0.25	0.41
	V_ss_	L/kg	0.28 ± 0.08	0.21	0.34
Pantoprazole	C_max_	ng/mL	2604 ± 381	1855	3412
(SC)	T_max_	hr	0.55 ± 0.21 (median: 0.63)*	0.33	0.75
	AUC_last_	hr*ng/mL	11.18 ± 9.81	5.37	23.10
	AUC_inf_	hr*ng/mL	11.70 ± 10.07	5.40	25.19
	AUMC_inf_	hr*ng/mL	52.04 ± 11.60	12.17	240.09
	MRT_inf_	hr	4.45 ± 3.72	2.25	9.53
	T_1/2_ (λz)	hr	2.48 ± 2.17	1.53	6.61
	λz	1/hr	0.23 ± 0.19	0.1	0.45
	V_z_/F	L/kg	0.37 ± 0.04	0.32	0.40
	F	(%)	83.33 ± 42.57	52.81	146.33

C0: calculated concentration at time zero of IV administration; AUC_last_: area under the curve calculated at the last time point; AUC_inf_: area under the curve extrapolated to infinity; AUMC_inf_: area under the moments curve extrapolated to infinity; MRT_inf_: mean residence time extrapolated to infinity; CL: plasma clearance; λz: elimination rate; T_1/2_ (λz): elimination half-life; Vz: volume of distribution: Vss: volume of distribution at steady-state; Cmax: maximum plasma concentration after SC administration; Tmax: time to reach maximum plasma concentration after SC administration; Vz/F: Volume of distribution accounting for bioavailability; F: bioavailability. Means are geometric with the exception of elimination half-life which is reported as harmonic mean. *Note: this median value would correspond to a time point between the 30 and 45 minute sample collections.

**Table 4:** Pharmacokinetic parameters of pantoprazole sulfone after single dose intravenous (IV) and subcutaneous (SC) administration of pantoprazole sodium (1.0 mg/kg) in sheep (n = 4).

Compound (Route)	Parameter	Unit	Mean ± SD	Min	Max
Pantoprazole	C_max_	ng/mL	281.5 ± 111.6	187	421
Sulfone	T_max_	hr	1.36 ± 0.25 (median: 1.5)	1	1.5
(IV)	AUC_last_	hr*ng/mL	2166.6 ± 923.4	1048.67	3176.18
	AUC_inf_	hr*ng/mL	2347.41 ± 910.61	1196.51	3277.6
	MRT_inf_	hr	5.73 ± 1.84	3.93	8.06
	T_1/2_ (λz)	hr	4.66 ± 1.78	3.8	7.57
Pantoprazole	C_max_	ng/mL	229.25 ± 102.19	181	394
Sulfone	T_max_	hr	2.45 ± 1.11 (median: 2.5)	1.5	4
(SC)	AUC_last_	hr*ng/mL	1802.67 ± 575.98	1101.56	2480.5
	AUC_inf_	hr*ng/mL	2057.52 ± 851.62	1149.19	3217.78
	MRT_inf_	hr	8.23 ± 5.75	4.42	16.57
	T_1/2_ (λz)	hr	4.13 ± 3.97	2.36	10.87

Cmax: maximum plasma concentration after SC administration; Tmax: time to reach maximum plasma concentration after SC administration; AUC_last_: area under the curve calculated at the last time point; AUC_inf_: area under the curve extrapolated to infinity; MRT_INF_: mean residence time extrapolated to infinity; T_1/2_ (λz): elimination half-life.

## Discussion

Our analysis focused on the pharmacokinetics of pantoprazole and the sulfone metabolite as well as the pharmacodynamics of pantoprazole with respect to abomasal pH. To our knowledge this is the first study evaluating the pharmacokinetics and pharmacodynamics of pantoprazole administered by IV and SC routes in sheep.

Similar studies have evaluated intravenous pantoprazole in calves, goats, and alpacas. Comparatively, the AUC_inf_ of the sheep in our study (15230 hr*ng/ml) is greater than the calves (13440 hr*ng/mL) and goats (1100 hr*ng/mL). The elimination half live for sheep after IV administration of pantoprazole (3.29 hr) was longer than reported for alpacas (0.47 hr), 2 day old calves (2.87 hr), approximately 1 month old calves (1.81 hr) and goats (0.7 hr) [8, 14–16]. Sheep have a lower bioavailability (83.33%) than alpacas (115%) and calves (115.2%) for subcutaneous administration. Comparing the sheep SC dosing to calves and alpacas, the AUC_inf_ of sheep (11,700 hr*ng/mL) is also greater than calves (7857 hr*ng/mL) and alpacas (2900 hr*ng/mL). Additionally, sheep have a longer half-life when given SC pantoprazole (2.48 hr) when compared to calves (1.81 hr) and alpacas (0.43 hr). Also of note is the half-life observed for pantoprazole exhibited a range (1.95–7.78 hours after IV administration, and 1.53–6.61 hours after SC administration) that could warrant further exploration, as additional variation may exist. This variation is also noted in the human proton pump inhibitor literature. For example, pantoprazole is noted to have a half-life range of 0.8–2.0 hr in people, with a clearance ranging between 90–225 mL/min [19] (human clearance PK values are not weight adjusted as veterinary are). It is important to note that the calves and alpacas were treated with twice the dose of pantoprazole than the sheep (1.0 mg/kg vs 2.0 mg/kg) of this study. These comparisons can be found in **Table 5**.

**Table 5:** Comparative pantoprazole pharmacokinetic values currently reported in the veterinary literature.

Species	Dose (route)	C0/Cmax (ng/mL)	T1/2λz (hr)	AUC (ng/mL*hr)	Bioavailability (F, %)	Reference
Alpacas	1.0 mg/kg (IV)	--	0.47	1420	NA	Smith et al, 2010 [8]
Alpacas	2.0 mg/kg (SC)	2840 (Cmax)	0.43	2900	115.0	Smith et al, 2010 [8]
Calves– 2 days old	1.0 mg/kg (IV)	4070 (Cmax)	2.87	13440	NA	Olivarez et al, 2020 [14]
Calves ~ 1 month old	1.0 mg/kg (IV)	2147 (C0)	1.44	3586	NA	Olivarez et al, 2023 [15]
Calves ~ 1 month old	2.0 mg/kg (SC)	3435 (Cmax)	1.81	7857	115.2	Olivarez et al, 2023 [15]
Goats	1.0 mg/kg (IV)	3100 (C0)	0.7	1100	NA	Smith et al, 2021 [16]
Sheep	1.0 mg/kg (IV)	6085 (C0)	3.29	15230	NA	Current Study
Sheep	1.0 mg/kg (SC)	2604 (C_max_)	2.48	11700	83.33	Current Study

Similar studies investigating intravenous pantoprazole have evaluated the kinetics of the metabolite pantoprazole sulfone in calves and goats. While the clinical significance of the metabolite is not currently fully elucidated, in calf studies, only the sulfone metabolite was detectable in tissue [14]. As such, information regarding the kinetics of the metabolite could provide useful for future investigations of residue studies, as sheep are a food animal species. With respect to pantoprazole sulfone, sheep of this study had a C_max_ (281.5 ng/mL) between calves (153 ng/mL) and goats (500 ng/mL). The AUC_inf_ of pantoprazole sulfone in the sheep (2347 hr*ng/mL) is between calves (2743 hr*ng/mL) and goats (200 hr*ng/mL). Sheep had a half-life (4.66 hr) between calves (10.5 hr) and goats (0.8 hr) for the sulfone metabolite. Comparing the sheep SC dosing to calves, the C_max_ of the metabolite in calves (383 ng/mL) is greater than sheep of this study (229.25 ng/mL). The half-life of calves (12.8 ng/mL) is greater than sheep (4.13 ng/mL) using a SC dose. The AUC_inf_ of the sulfone metabolite in SC dosing of calves (7086 hr*ng/mL) was greater than in sheep (2057.52 hr*ng/mL). Calves have a greater T_max_ (2.77 hr) of the sulfone metabolite when compared to sheep (2.45 hr). It is important to note that sheep were given half the dose of SC pantoprazole than calves.

Our study was not designed to determine the interspecies differences in the PK of pantoprazole. While sheep, cattle and goats are domestic ruminants with many physiologic similarities, there are some differences that could explain some of the PK differences. Hepatic oxidative metabolism has been demonstrated to be more rapid in sheep and goats than cows [20]. Allometric scaling could also provide an explanation for differences between animals of similar physiology, but difference sizes, such as cattle and sheep [21]. Future studies could evaluate this variation amongst ruminant species for similar drugs.

In our study sheep administered pantoprazole IV or SC had a pH above 4 for at least 8–12 hr after administration. Comparatively this is important as in other species such as horses and humans it is thought that a gastric pH higher than 4.0 is ideal for mucosal healing [22]. The length of time that abomasal pH remained above 4.0 in this study was longer than what was observed in a similar study that evaluated the pharmacokinetics and pharmacodynamics of esomeprazole in sheep [17]. In that study abomasal pH remained above 4.0 for at least 8 hours after IV administration of 1.0 mg/kg of esomeprazole. Another proton pump inhibitor, omeprazole has been investigated after oral administration to sheep and found to have no effect on abomasal pH after oral administration [23].

An interesting finding of this study was the observation that the ewes administered 1.0 mg/kg of pantoprazole SC had statistically significant decreases to abomasal pH. Previous ruminant studies have used a 2.0 mg/kg SC dose [8, 15]. This decreased dosage still having a clinical effect may be useful for livestock practice in ruminants, where economics can sometimes be a restricting factor for treatment.

Limitations of this study include the small sample size. While a study population of 4 animals may not capture all variation within a population, this sample size has been used in other ruminant pharmacokinetic/pharmacodynamic studies of gastroprotectants [17, 24]. An additional limitation is the single dose pharmacokinetics. As such, future studies should evaluate the pharmacokinetics and pharmacodynamics of pantoprazole in larger populations and evaluate multiple-dose pharmacokinetics. Clinical safety was not exhaustively evaluated by this study, however a previous retrospective study of the use of pantoprazole in hospitalized ruminants did not find evidence of the same adverse effects found in people when pantoprazole is used [13].

There are many future directions for this work. Investigating pantoprazole in an abomasal ulcer challenge model would be useful for determining clinical efficacy. Recently, oral omeprazole was shown to be ineffective in controlling abomasal ulceration in a phenylbutazone-induced ulcer model in sheep [7]. This model could be utilized to test oral administration of pantoprazole in sheep. While the study population was one of low variability, a larger population could be utilized to further investigate factors that could influence the pharmacokinetics of pantoprazole in sheep, potentially utilizing non-linear mixed effects to explore these relationships [25].

It is important to note that the administration of pantoprazole as a gastroprotectant in sheep is an extra-label use of this medication and requires a valid veterinarian-client-patient relationship with appropriate veterinary oversight. In the United States or Canada, veterinarians could contact the Food Animal Residue Avoidance Databank (FARAD), or Canadian Global Food Animal Residue Avoidance Databank (CGFARAD) for extra-label use withdraw period recommendations after using this product in sheep. Pantoprazole and its sulfone metabolite have been demonstrated to be rapidly eliminated from the edible tissues of calves after single IV administration [14], but no tissue residue information is currently available for sheep administered pantoprazole by the IV or SC routes.

Conclusions: This study reports the pharmacokinetics and pharmacodynamics of a 1.0 mg/kg dose of pantoprazole administered to sheep IV and SC. Pharmacokinetic parameters are similar to those reported for other large animal species after the first administration, with characteristics such as a short elimination half-life, high initial concentration and high subcutaneous bioavailability. The bioavailability of the SC administration was approximately 83% in the study sheep, which could provide an economical method for use in the field as a higher SC dose may not be necessary to achieve similar systemic exposure to IV. Both routes of administration increased abomasal pH above 4.0 for a minimum of 12 hours after administration, supporting the use of pantoprazole for abomasal ulceration in sheep. Future work should focus on evaluating the efficacy of pantoprazole in experimental models or naturally occurring abomasal ulceration in sheep.

## Abbreviations

IV: Intravenous
SC: Subcutaneous
UV: Ultraviolet
